# Evaluating palliative opportunities in pediatric patients with leukemia and lymphoma

**DOI:** 10.1002/cam4.3862

**Published:** 2021-03-22

**Authors:** Emily J. Labudde, Nicholas P. DeGroote, Susie Smith, Jonathan Ebelhar, Kristen E. Allen, Sharon M. Castellino, Karen Wasilewski‐Masker, Katharine E. Brock

**Affiliations:** ^1^ Emory University School of Medicine Atlanta GA USA; ^2^ Aflac Cancer and Blood Disorders Center of Children's Healthcare of Atlanta Atlanta GA USA; ^3^ Department of Pediatrics Division of Pediatric Hematology/Oncology Emory University Atlanta GA USA; ^4^ Department of Pediatrics Division of Pediatric Palliative Care Emory University Atlanta GA USA

**Keywords:** end‐of‐life, leukemia, lymphoma, oncology, palliative opportunity, pediatric palliative care

## Abstract

**Background:**

Despite favorable prognoses, pediatric patients with hematologic malignancies experience significant challenges that may lead to diminished quality of life or family stress. They are less likely to receive subspecialty palliative care (PC) consultation and often undergo intensive end‐of‐life (EOL) care. We examined “palliative opportunities,” or events when the integration of PC would have the greatest impact, present during a patient's hematologic malignancy course and relevant associations.

**Methods:**

A single‐center retrospective review was conducted on patients aged 0–18 years with a hematologic malignancy who died between 1/1/12 and 11/30/17. Demographic, disease, and treatment data were collected. A priori, nine palliative opportunity categories were defined. Descriptive statistics were performed. Palliative opportunities were evaluated over temporal quartiles from diagnosis to death. Timing and rationale of pediatric PC consultation were evaluated.

**Results:**

Patients (*n* = 92) had a median of 5.0 (interquartile range [IQR] 6.0) palliative opportunities, incurring 522 total opportunities, increasing toward the EOL. Number and type of opportunities did not differ by demographics. PC consultation was most common in patients with lymphoid leukemia (50.9%, 28/55) and myeloid leukemia (48.5%, 16/33) versus lymphoma (0%, 0/4, *p* = 0.14). Forty‐four of ninety‐two patients (47.8%) received PC consultation a median of 1.8 months (IQR 4.1) prior to death. Receipt of PC was associated with transplant status (*p* = 0.0018) and a higher number of prior palliative opportunities (*p* = 0.0005); 70.3% (367/522) of palliative opportunities occurred without PC.

**Conclusion:**

Patients with hematologic malignancies experience many opportunities warranting PC support. Identifying opportunities for ideal timing of PC involvement may benefit patients with hematologic cancers and their caregivers.

## Graphical abstract

Palliative opportunities are events throughout a child's course with cancer that warrant additional palliative care support. Children with hematologic malignancies experienced a median of five palliative opportunities throughout their course, which increase toward death, yet received palliative care consultation only 47% of the time.

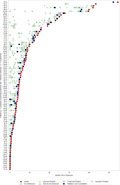

## 1 INTRODUCTION

Pediatric patients with hematologic malignancies comprise 40% of childhood cancers,^1^ and have been the largest beneficiaries of curative approaches in pediatric cancer with 5‐year disease‐free survival greater than 80%, and efficacious salvage therapies for those who relapse.^1^ Parents and providers of children with hematologic malignancies maintain hope for cure, even amidst relapsed disease when the probability of cure decreases.^2^, ^3^, ^4^ Compared to the caregivers of children with solid tumors, these parents acknowledge that cure is unrealistic much closer to death.^5^ Children with hematologic malignancies have different disease and end‐of‐life (EOL) experiences,^6^ receiving more cure‐directed therapy, intensive care at EOL, and longer, more frequent hospital or intensive care unit (ICU) admissions.^7^, ^8^, ^9^, ^10^, ^11^ These patients are more likely to die from therapy‐related complications, receive less hospice support, and more often die in the ICU.^7^, ^10^, ^12^, ^13^, ^14^, ^15^ Consequently, patients often experience greater suffering at the EOL and bereaved parents report wishing they had integrated palliative goals earlier.^2^, ^3^, ^15^

Despite high cure rates, children with cancer and their families experience challenges related to symptom management, emotional, and psychosocial wellbeing, and EOL decisions.^15^, ^16^, ^17^, ^18^, ^19^, ^20^ Integrating subspecialty palliative care (PC) into cancer care can improve the quality of life (QOL)^14^, ^15^, ^19^, ^21^, ^22^, ^23^ and elongate survival.^24^ Despite improved access to subspecialty pediatric PC^25^ and guidelines supporting PC as a standard of care for children with cancer,^19^, ^26^ children with hematologic malignancies receive less and later PC, often when disease and symptoms are more advanced.^9^, ^27^, ^28^

Optimal timing for PC integration into pediatric cancer care has been difficult to determine. Parents are accepting of PC involvement at diagnosis, even when the goal is cure, because burdensome symptoms begin early in cancer therapy.^17^, ^18^, ^29^ Conversely, late integration of PC is associated with under‐documented goals of care, duration of cancer‐specific therapy beyond the point of benefit, hospital admission near the EOL, and death in the ICU.^9^, ^17^, ^30^ Pediatric oncologists recognize that PC is beneficial at all stages of treatment, regardless of prognosis, and that early integration maximizes benefit.^21^, ^31^ Yet, in practice, oncologists often wait for a precipitating event or EOL trigger to introduce PC.^14^, ^22^, ^31^, ^32^, ^33^ Although the time from PC consult to death is increasing,^17^ referrals and timing continue to be variable across diagnoses and oncology teams.^28^

Many opportunities for PC consultation occur throughout a child's cancer course. A “palliative opportunity” has been defined as an event during a patient's disease course at which time subspecialty PC, or care provided by clinicians with subspecialty training or board certification in PC, could be provided to diminish suffering and improve the patient's or family's overall experience.^34^ The primary aim of this study was to examine the number and timing of palliative opportunities between diagnosis and death in children with hematologic malignancies who did not survive. We hypothesized that patients with myeloid leukemia have a higher number of palliative opportunities, and demographic variables influence the number of palliative opportunities. The secondary aim was to assess PC consultation, hypothesizing that patients who received PC consultation experienced a higher number of palliative opportunities than patients not receiving PC, and received their consultation due to progressive disease.

## 2 METHODS

### 2.1 Study design

This retrospective chart review was conducted at Children's Healthcare of Atlanta (CHOA). All patients aged 0–18 years at diagnosis who received care at CHOA, with a primary diagnosis of leukemia or lymphoma, and who died between January 1, 2012 and November 30, 2017 were identified. Inpatient pediatric PC services became available in 2011. Patients were excluded if they were lost to follow‐up or received care outside of CHOA without adequate electronic health record (EHR) documentation of care received. Patients with initial diagnosis and/or treatment occurring prior to the implementation of the EHR in 2006 were excluded if adequate documentation of their prior course was not available. The CHOA Institutional Review Board granted exempt status.

### 2.2 Defining palliative opportunities

Prior to data collection, a list of “palliative opportunities” was established through an expert panel including subspecialty PC, pediatric oncology, and pediatric palliative oncology physicians.^34^ Palliative opportunities were defined as events during a patient's cancer course with an increased risk of morbidity or distress, as viewed by the patient or family, in which PC could be initiated or intensified. A list of nine palliative opportunities, applicable across all pediatric cancers, was defined (Table 1). Palliative opportunities were grouped into categories: (1) disease‐related; (2) treatment‐related; (3) symptom‐related; (4) ICU‐related; or (5) EOL‐related (Table 1). Of note, initial diagnosis was not included as an event, and only symptoms leading to hospital admission were included, rather than those arising *during* a hospital course.

**TABLE 1:** Palliative opportunities in pediatric patients with cancer

Opportunity	Category
1. Progression of disease	Disease
2. Relapse of disease	Disease
3. Hematopoietic Stem Cell Transplant (HSCT) or Chimeric Antigen Receptor T‐cell therapy (CAR‐T)	Treatment
4. Enrollment in phase 1 trial	Treatment
5. Hospital admission for severe symptoms	Symptom
Pain or dyspnea requiring intravenous (IV) opioids	
Nausea/vomiting requiring IV anti‐emetics	
Fatigue	
Progressive neurologic symptoms	
Social concerns	
6. Intensive care unit (ICU) admission	Intensive care
7. Admission for end‐of‐life care	End‐of‐life
8. Placement of do‐not‐resuscitate (DNR) order	End‐of‐life
9. Enrollment in hospice	End‐of‐life

### 2.3 Data collection

Demographic information (age at diagnosis, age at death, sex, race, ethnicity, religion, primary parental language, insurance status), disease‐related information (type of hematologic malignancy, relapse, progression, hospital admission, hematopoietic stem cell transplant [HSCT], chimeric antigen receptor T‐cell [CAR‐T] therapy, phase 1 trial enrollment), EOL information (do not resuscitate [DNR] order, hospice enrollment, cause of death), and the date and reason for PC consultation were obtained systematically using a standardized data abstraction guide via manual EHR chart review including evaluation of clinic, admission and consult notes, physical exam findings, imaging, and pathology results. The date of each palliative opportunity was recorded. Each event matching a palliative opportunity was counted as a unique opportunity unless a patient was admitted with multiple symptoms or symptoms to the ICU, which weres only recorded once.

The reason for PC consultation was determined from the initial PC consultation note and categorized as disease‐related (progression, relapse), symptom management (pain, dyspnea, fatigue, nausea/vomiting), or EOL‐related (DNR, hospice enrollment, EOL management), as defined in Table 3. The palliative opportunity immediately preceding consultation was obtained.

### 2.4 Statistical analysis

Descriptive statistics included frequencies and percentages for categorical variables and means (standard deviation, SD) or medians (interquartile range, IQR) for continuous variables. Independent two‐sample *t*‐test or ANOVA was used to determine if the number of palliative opportunities differed by demographics, primary diagnosis, or having received a PC consultation. Chi‐square or Fisher's exact test was used to evaluate for the association between diagnosis and the reason for PC consultation or the preceding palliative opportunity. The timing of palliative opportunities was also assessed by evaluating a patient's disease course over quartiles from diagnosis to death. *p*‐values were two‐sided and considered significant if *p* < 0.05. All analyses were conducted using SAS Enterprise Guide, v.7.1.

## 3 RESULTS

A total of 112 patients with leukemia or lymphoma died between January 1, 2012 and November 30, 2017. Ninety‐two (82.1%) patients were included for analysis. Twenty patients were excluded due to: lost to follow‐up (*n* = 8), gap in care (*n* = 2) or transferred care without adequate outside records (*n* = 9), or treatment prior to EHR (*n* = 1).

Demographic information is summarized in Table 2. The median age at diagnosis was 7 years (range 0–18) with 23.9% diagnosed prior to age two. The median age at death was 11 years (range 0–20). Patients were predominantly male (55.4%), white (52.2%), non‐Hispanic (80.4%), English‐speaking (86.7%), identified as Christian (94.6%), and had Medicaid insurance (55.4%). The most common diagnosis was lymphoid leukemia (*n* = 55, 59.8%), followed by acute/chronic myeloid leukemia (*n* = 33, 35.6%) and Hodgkin/Non‐Hodgkin lymphoma (*n* = 4, 4.4%). Patients had a median of 1.0 (IQR 3.0, range 0–9) progression and relapse events. Forty‐two patients (45.7%) died without ever having a relapse/progression event. Thirty‐five patients (38.0%) underwent HSCT, four (4.4%) received CAR‐T therapy, and 18 (19.6%) enrolled in a phase I trial. In assessing EOL care, 25 (27.2%) patients enrolled in hospice a median of 34 days prior to death, 69 (75.0%) had a DNR order documented a median of 2.0 days (IQR 12.0) before death, and 1 (1.1%) patient was admitted to the hospital specifically for EOL care. Fifty‐two percent (48/92) of patients had an ICU admission in the last month of life. The most common cause of death was disease progression (53%, 49/92).

**TABLE 2:** Demographic characteristics of patients with hematologic cancers who died

	Median (IQR)	*N* = 92 *n* (%)
Age		
Age at diagnosis (years)	7.0 (11.0)	
Age at death (years)	11.0 (11.5)	
Sex		
Female		41 (44.6)
Male		51 (55.4)
Race		
White		48 (52.2)
Black		38 (41.3)
Asian/Southeast Asian		4 (4.4)
Unknown		2 (2.1)
Ethnicity		
Non‐Hispanic		74 (80.4)
Hispanic^a^		18 (19.6)
Language		
English		78 (86.7)
Religion		
Christian^b^		77 (83.7)
Jewish		2 (2.2)
Muslim		2 (2.2)
Unknown		11 (12.0)
Diagnosis		
Lymphoid leukemia^c^		55 (59.8)
Myeloid leukemia^d^		33 (35.6)
Lymphoma^e^		4 (4.4)
Insurance		
Medicaid		51 (55.4)
Private insurance		15 (16.3)
Tricare (military)		4 (4.4)
Other insurance		16 (17.4)
Uninsured		6 (6.5)

^a^ Hispanic includes Mexican NOS, Mexican Chicano, Puerto Rican, South or Central American (except Brazil), Hispanic, NOS, Spanish, NOS, or Latino, NOS.

^b^ Christian denominations include Unitarian and Catholicism.

^c^ Includes B‐cell and T‐cell acute lymphoblastic leukemia (ALL) and lymphoid lymphomas (treated as leukemia).

^d^ Includes acute myeloid leukemia (AML), myeloid sarcoma, and chronic myelogenous leukemia (CML).

^e^ Includes Hodgkin, Burkitt, anaplastic large cell, and diffuse large B‐cell lymphomas.

**FIGURE 1:**
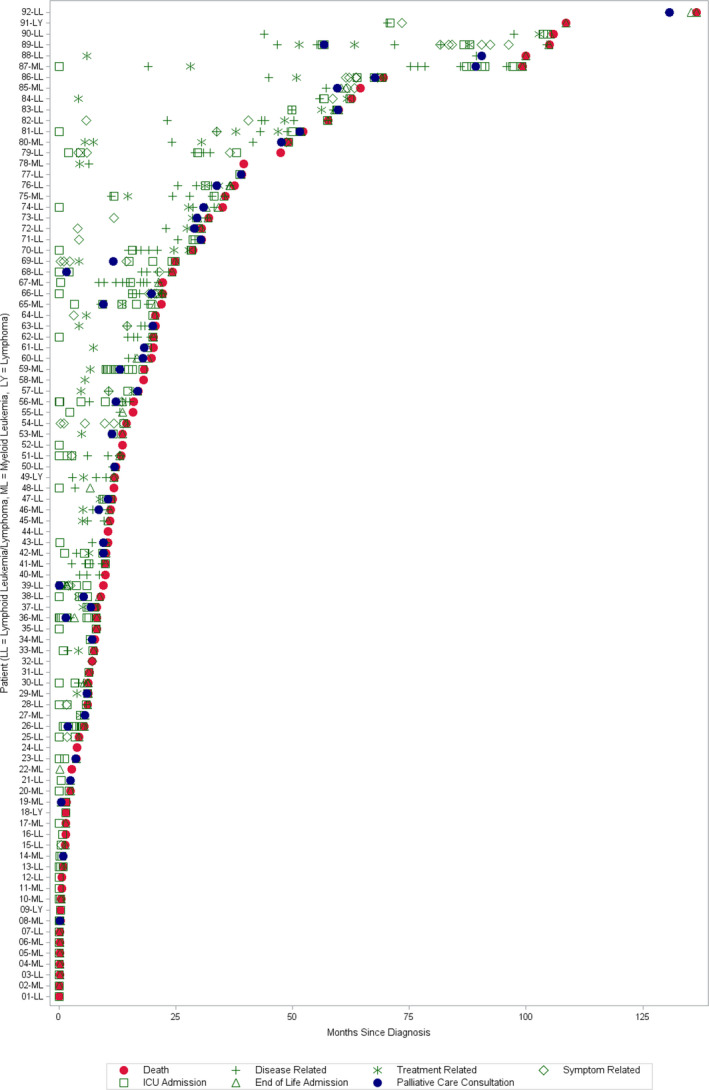
Patient‐specific palliative opportunities from the time of diagnosis to death. LL, lymphoid leukemia; LY, lymphoma; ML, myeloid leukemia/lymphoma

Overall, a total of 522 palliative opportunities were identified among these 92 patients. Patients had a median of 5.0 (IQR 6.0, range 0–21) palliative opportunities between diagnosis and death (median 11.2 months). The first palliative opportunity occurred at a median of 0.8 months (IQR 5.1) after diagnosis. A summative view of each patient's disease trajectory defined by each palliative opportunity is seen in Figure 1. When the disease course was divided into temporal quartiles, palliative opportunities increased toward the EOL, with a median of 1.0 (IQR 1.0), 0.0 (IQR 1.0), 0.0 (IQR 2.0), and 2.5 (IQR 4.0) palliative opportunities during the first, second, third, and fourth quartiles, respectively. As race, ethnicity, social determinants of health, and Medicaid insurance have been associated with symptom burden, PC consultation, and EOL outcomes in prior adult and pediatric oncology studies, demographics were assessed in relation to palliative opportunities.^35^, ^36^, ^37^, ^38^, ^39^ The total number of palliative opportunities did not differ by sex (*p* = 0.12), race (*p* = 0.75), ethnicity (*p* = 0.61), age at diagnosis (*p* = 0.67), primary language (*p* = 0.68), primary insurer (*p* = 0.84), or primary cancer diagnosis (*p* = 0.46). Type of opportunities did not differ across diagnosis groups, except that patients with lymphoblastic disease exhibited a wider range of symptom‐related opportunities (0–6) than those with myeloblastic disease (0–2) or Hodgkin/Non‐Hodgkin lymphoma (0–1), (*p* = 0.02) despite similar medians across primary diagnosis groups.

In total, 47.8% (44/92) of patients received subspecialty PC consultation. By primary diagnosis, 50.9% (28/55) of patients with lymphoid leukemia, 48.5% (16/33) of patients with myeloid leukemia, and 0% (0/4) of patients with Hodgkin/Non‐Hodgkin lymphoma received PC consultation (*p* = 0.14, Table 3). Patients receiving PC had a higher total median number of palliative opportunities (6.5, IQR 6.5) than those who did not (3.0, IQR 4.0) (*p* = 0.0005). Patients experienced a median of three palliative opportunities (IQR 4.0) prior to PC consultation. Amongst all patients, 70.3% (367/522) of palliative opportunities occurred without PC support in place.

**TABLE 3:** Palliative opportunities and palliative care consultation stratified by diagnosis

	Lymphoid leukemia (*N* = 55)	Myeloid leukemia (*N* = 33)	Lymphoma (*N* = 4)	*p*‐value
Palliative opportunity categories, median (IQR)^a^				
Disease‐related	1.0 (3.0)	1.0 (2.0)	0.5 (2.0)	0.84
Treatment‐related	0.0 (1.0)	1.0 (1.0)	0.0 (1.0)	0.85
Symptom‐related	0.0 (1.0)	0.0 (0.0)	0.5 (1.0)	0.02
End‐of‐life‐related	1.0 (0.0)	1.0 (0.0)	0.5 (1.0)	0.27
Intensive care‐related	1.0 (2.0)	1.0 (1.0)	1.0 (0.5)	0.48
Total opportunities	5.0 (5.0)	4.0 (6.0)	2.5 (4.5)	0.46
Palliative care consultation, *n* (%)				
Yes	28 (50.9)	16 (48.5)	0 (0.0)	0.14
No	27 (49.1)	17 (51.5)	4 (100.0)	
Reason for palliative care consultation, *n* (%)^b^				
Disease‐related	18 (32.7)	12 (36.4)	0 (0.0)	0.62
Symptom management	5 (9.1)	2 (6.1)	0 (0.0)	
End‐of‐life‐related	5 (9.1)	2 (6.1)	0 (0.0)	

Abbreviations: DNR, do not resuscitate; EOL, end of life; IQR, interquartile range.

^a^ Palliative opportunity categories as noted in Table 1.

^b^ Reason for palliative care consultation was similarly categorized as disease‐related (progression, relapse), symptom management (pain, dyspnea, fatigue, nausea/vomiting), and end‐of‐life (EOL) related (DNR, hospice enrollment, EOL management).

Longer survival time from diagnosis to death was noted for patients receiving PC compared to those that did not (median 18.9 months vs. 7.3 months). Among those receiving PC, the median time from diagnosis and first opportunity to PC involvement were 12.0 and 6.2 months (IQRs 26.6 and 12.3), respectively. Most (77.2%, 33/44) PC consultations occurred in the last quartile of the disease, or a median of 1.8 months (IQR 4.1) prior to death.

The most common documented reason for PC consultation was disease‐related progression/relapse (30, 68.2%), followed by EOL‐related (7, 15.9%), and symptom management (7, 15.9%, Table 3). The palliative opportunity that immediately preceded PC consultation (median 14.5 days) was relapse/progression (14, 31.8%), ICU admission (15, 34.1%), DNR order (5, 11.4%), HSCT/CAR‐T (5, 11.4%), and hospice enrollment (5, 11.4%). PC consultation was more common amongst patients who received HSCT (24/35, 68.6%, *p* = 0.0018) and among those who survived more than 1 year from diagnosis (27/43, 62.7%, *p* = 0.0071). Patients who received PC consultation were more likely to have enrolled in hospice (19/44 [43.2%] vs. 6/48 [12.5%], *p* = 0.001). Most patients with DNR orders had the limits on resuscitation placed very near the EOL (4 days [range 0–242 days] in those receiving PC vs. 1 day [range 0–79 days] in those without PC, *p* = 0.07).

## 4 DISCUSSION

Differences in therapy, prognosis, and therapy‐associated morbidity underlie differences in PC needs for children with hematologic malignancies compared to other oncologic diagnoses. Understanding the best time for PC integration and its impact on the child's experience can enhance care delivery and reduce suffering. In the largest evaluation of patterns of palliative opportunities and PC consultation focused on children with hematologic malignancies, we identified that palliative opportunities were common in our cohort and increase toward the EOL. However, less than half of patients received PC consultation, often late in their disease course.

Patients in our cohort experienced a median of five palliative opportunities throughout their disease course, compared to a mean of nine opportunities in children with sarcomas at the same institution.^34^ There was no association between the number of palliative opportunities and demographic factors or primary diagnosis. A lack of religious diversity precluded the examination of the association between religion and palliative opportunities.

Despite the growing acceptance of and access to PC in pediatric oncology, only 47.8% of these patients received subspecialty PC. While this percentage is higher than in other pediatric studies involving deceased cohorts,^9^, ^28^, ^40^, ^41^, ^42^ missed opportunities and late consultation remained common, with 70.3% of palliative opportunities occurring prior to or without PC involvement. Among older adults with hematologic cancer, early PC was associated with increased hospice utilization and decreased health care use at EOL.^43^ In our pediatric cohort, PC consultation occurred a median of 1.8 months before death. Even when survival time is short, PC consult should ideally occur *before* the bulk of palliative opportunities arise, such that a therapeutic alliance has formed before physical, psychological, social, or existential suffering occurs.^31^

Patients with B/T‐cell lymphoblastic leukemia/lymphoma experienced five palliative opportunities, compared to four among patients with myeloblastic disease and 2.5 in patients with lymphoma. Importantly, a good prognosis does not always lessen the risk of highly stressful events. Palliative opportunities in patients with myeloblastic leukemia could have been underrepresented given that the standard of care is hospitalization for the duration of a treatment course; symptoms arising during their hospitalization were not counted.

We hypothesized that patients experiencing more palliative opportunities would be more likely to receive PC consultation due to progressive symptom and disease burden, compassion fatigue from the primary team, and need for goals of care discussion. This was confirmed in our cohort, as children who received PC experienced twice the number of palliative opportunities compared to patients who did not receive PC. Survival time and HSCT were possible drivers of this relationship. Patients surviving less than 1 year from initial diagnosis received PC 34.7% of the time compared to 62.7% of patients surviving longer than 1 year. Some patients with hematologic malignancy likely had good upfront prognoses until a sudden adverse event occurred, making PC consult less likely in those patients. HSCT is an intensive therapy with risk for morbidity and death that could benefit from early PC integration.^44^ In our institution, PC is often consulted post‐HSCT, generally related to complications such as graft versus host disease, relapse, and discussion around goals of care related to additional post‐transplant therapies. HSCT patients received PC twice as often as non‐HSCT patients (68.6% vs. 31.4%). HSCT patients spend long periods of time hospitalized, potentially missing capture of palliative opportunities that arose during this prolonged hospitalization but allowing more time for inpatient PC consultation. While not yet known in pediatrics, integrated PC for adult HSCT recipients temporarily improves QOL and lessens depression, symptom burden, and post‐traumatic stress.^45^, ^46^ Although few patients received CAR‐T, this therapeutic advancement is occurring with increasing frequency.^47^ Additional studies could assess how CAR‐T affects palliative opportunities or PC consultation.

In this cohort of children with hematologic cancers, patients often received intensive therapies towards the EOL. First, over half of patients were admitted to the ICU in the last month of life, consistent with studies demonstrating high frequency of ICU admissions in patients with hematologic malignancies.^7^, ^8^, ^9^, ^10^, ^11^ ICU admission was the preceding opportunity one‐third of the time before PC consultation, suggesting intensivists, rather than oncologists, requested PC consultation. Second, 10% of patients died from therapy‐related complications, more common in patients with hematologic malignancies due to the relatively better prognosis, prevalence of HSCT and chemotherapy towards the EOL, and pursuit of cure.^8^, ^11^, ^15^ Third, DNR orders were entered a median of 2 days before death, reflective of a continued focus on cure, delayed goals of care conversations, and transitioning toward comfort‐focused care when the patient is actively dying. Patients with hematologic malignancies often have the later establishment of DNR orders, in contrast to patients with other cancers^12^; training of primary oncologists and involvement of PC before EOL could facilitate earlier advance care planning discussions. While the difference in time from DNR order to death between patients with or without PC support did not achieve significance (4 days vs. 1 day, respectively), an additional 3 days is *clinically* significant. This additional time permits a shift of focus from treatment towards comfort‐focused care, reduces family and provider distress, facilitates memory‐making activities, and, if desired, allows time for discharge home with hospice or family visitation before the patient dies. Lastly, only 27.2% of patients received hospice care, far below the 71.7% of patients with sarcomas at this institution who received hospice.^34^ Most hospice recipients had received PC; even among all patients who received PC, less than half received hospice care, consistent with prior data showing patients with hematologic malignancies are less likely to receive hospice or die at home.^7^, ^8^, ^12^ This could be due to the trend of late PC consultation, family preference for inpatient EOL care, continued attempts at curative therapy, or death during a high‐risk event when hospice would not have been recommended. As all patients were younger than age 21 with over half of the patients having Medicaid insurance, they would have been eligible for Concurrent Care (simultaneous hospice and hospital‐based care).^48^ Therefore, continued chemotherapy or transfusion needs should not have posed a barrier to hospice enrollment. Comparison to patients with other cancers may improve understanding of intensive care among patients with hematologic malignancies.

Limitations include being performed at a large, tertiary care, single institution with an inpatient PC team. This may not be generalizable to all pediatric oncology centers. All patients were deceased, so the number of met and unmet palliative opportunities in patients who survive their hematologic cancer is unknown. Data collection methods, notably around symptom‐related opportunities, underestimate the total number of opportunities in several ways: (1) admissions for two or more symptoms were not counted as multiple opportunities; (2) symptoms that arose during an admission for another reason (chemotherapy, HSCT, or fever) were not included; (3) an admission for pain lasting 1 day versus several weeks were both designated as one palliative opportunity, although the overall burden would be different. Young adults, who are frequently treated in pediatric centers, were excluded but are known to have high symptom burden, advance care planning needs, and distress.

Future directions for research include assessing EOL outcomes and patient QOL in relation to PC consultation timing, comparison of palliative opportunities based on primary cancer diagnosis, and development of protocols or EHR alerts to improve consistency and equity in PC consultation. This study quantified all palliative opportunities equally, though a weighted system, considering the intensity of the event and physical/psychosocial impacts, may better mimic the patient experience and inform how and when to consult PC to maximize benefit.

## 5 CONCLUSION

Patients with terminal hematologic malignancies experience numerous palliative opportunities, increasing toward the EOL. Multiple missed opportunities exist for PC involvement to discuss goals of care and improve QOL through relief of physical, psychological, and psychosocial symptoms. Understanding the physical and psychosocial burden associated with significant events in a patient's disease course as well as the benefits of PC involvement could lead to more consistent consultation, decreasing the prevalence of missed opportunities. Defining palliative opportunities allow providers to recognize the symptom burden and distress experienced by these patients and families and identify early opportunities for PC involvement.

## CONFLICT OF INTEREST

The authors have no relevant conflicts of interest to disclose.

## ETHICAL APPROVAL

Emory University and Children's Healthcare of Atlanta IRB determined this study to be exempt.

## Data Availability

The data that support the findings of this study are available from the corresponding author upon reasonable request.
